# Development of Prediction Models for Soil Nitrogen Management Based on Electrical Conductivity and Moisture Content

**DOI:** 10.3390/s22186728

**Published:** 2022-09-06

**Authors:** Hasan Mirzakhaninafchi, Indra Mani, Murtaza Hasan, Ali Mirzakhani Nafchi, Roaf Ahmad Parray, Dinesh Kumar

**Affiliations:** 1 Department of Farm Machinery & Power Engineering, College of Agricultural Engineering and Technology (COAE&T), Punjab Agricultural University (PAU), Ludhiana 141004, India; 2 Division of Agricultural Engineering, Indian Council of Agricultural Research-Indian Agricultural Research Institute (ICAR-IARI), New Delhi 110012, India; 3 Centre for Protected Cultivation Technology, Indian Council of Agricultural Research-Indian Agricultural Research Institute (ICAR-IARI), New Delhi 110012, India; 4 Precision Agriculture Extension, Raven Precision Agriculture Center, South Dakota State University, Brookings, SD 57007, USA; 5 Division of Agronomy, Indian Council of Agricultural Research-Indian Agricultural Research Institute (ICAR-IARI), New Delhi 110012, India

**Keywords:** algorithms, electrical conductivity, precision agriculture, sensors, nitrogen management

## Abstract

A study was conducted with the goal of developing an algorithm for use in sensors to monitor available soil N. For this purpose, three different soils were selected. The soils were studied for electrical conductivity (EC) at four different moisture levels and four levels of N. The selection of moisture levels was based on optimum moisture levels between tillage moisture and field capacity. The results revealed a significant relationship between electrical conductivity and moisture level of the soil as well as between electrical conductivity and soil N content. Based on these relations, a polynomial model was developed between the EC of each selected soil sample and moisture content as well as N levels. The regression model for moisture content-based EC determination had coefficients of determination of 0.985, 0.988, and 0.981 for clay loam, sandy loam, and sandy loam soils, respectively. Similarly, the regression model for N content-based EC determination had coefficients of determination of 0.9832, 0.9, and 0.99 for clay loam, sandy loam, and sandy loam soils, respectively. An algorithm developed using a polynomial relationship between the EC of each selected soil sample at all moisture and N levels can be used to develop a sensor for site-specific N application.

## 1. Introduction

As awareness of the need to reduce the use of costly inputs in precision farming has increased, the field of precision agriculture has also begun to draw the attention of planners, researchers, and users. Precision farming may be defined as an accurate application of agricultural inputs for crop growth considering relevant factors such as soil, weather, and crop management practices [1]. More specifically, it is an information and technology-based farming system where inputs are managed and distributed on a site-specific basis for long-term benefit. The concept of precision agriculture was developed in the 1990s and initially referred to the application of technologies that allowed for site-specific (spatial) management in a field-scale crop production context. Zhang and Kovacs [2] refer to precision agriculture as “the application of geospatial techniques and sensors to identify variations within a field and the solution to smooth the difference using diverse strategies”. Precision farming helps farmers to maximize the effectiveness of inputs. The major aims of precision agriculture technologies are to increase the profitability of crop production, improve product quality, and protect the environment, via site-specific management of agricultural inputs.

Variability in field conditions is one of the major contributors to field-scale differences in yield; therefore, varying agricultural inputs according to crop requirements could be beneficial. As such, information with respect to the variability of different soil attributes in the field is mandatory for any decision-making process. The ability to generate such information accurately at an affordable cost is a significant limitation in precision agriculture today. Fertilizer is both one of the major input costs for farmers and a source of great variability in the field. N available to plant roots in the soil is the main factor limiting the yield of crops. Variable rate application of fertilizers without accurate soil maps is often inappropriate and may result in economic losses. Hence, sensor development is expected to increase the effectiveness of variable application of fertilizers. Measurement of soil properties with the use of sensors has the potential to provide benefits at a relatively low price [3]. Fertilizer requirements vary within a field and in different cropping seasons based on the soil type, climate, and crops grown during previous years. Fertilizers are either expensive or their efficiency is limited based on the availability of water, especially in semi-arid and semi-humid zones. N is the main fertilizer being applied by farmers; therefore, improving N use efficiency is vital. To manage N fertilizer rates, it is essential to consider the organic N mineralized and soil inorganic N in crop development period [4]. In order to enhance crop productivity by controlling the N fertilizer rate, it is necessary to determine the exact N mineralized amount in soil organic matter (SOM). Soil mineral N measurement in real time is very difficult to achieve, as N is available to crop in the form of both nitrate (NO_3_^−^) and ammonium (NH_4_^+^) in soil [5,6]. Crops use different forms of N in soils; inorganic forms of N consist of nitrate, nitrite, and ammonium. Nitrate is the main form of N in many aerated soils; however, ammonium can be the main form in several acidic and/or anaerobic soils [7]. To improve the efficiency of crop production systems, affordable, reliable, and easily executable methods to measure the available mineral N in the top 0.90 m of soil need to be developed [8]. Aerobic incubation is one feasible way to predict the approximate amount of mineral N in soil; however, this takes a long time and only works in a fixed condition [9,10]. This technique is used to identify the capability of conversion of soil organic N to mineral, and to specify the feasibility of mineralizable organic N. This is the unstable N supply deemed to be a normal prediction of probable mineralization in soil and indicates the rate of N which is feasible to be mineralized [11,12].

Martínez and Galantini [10] conducted an experiment over an extended period of time to estimate organic N mineralization through a fast and simple approach using several chemical and biological procedures. However, laboratories reject the use of these procedures on soils as they require a long period of time to implement. For different soil monitoring techniques, organic indices, for instance, anaerobic incubation is critical [13,14]. In chemical procedures, the idea is that a particular solvent removes N from a pool. However, in these procedures, it is not essential for the removed N to be chemically homogeneous or biologically significant [12]. To evaluate the soil N mineralization, digestion of soil with a high salt can be applied [15]. Nevertheless, chemical approaches may have detrimental impacts on the environment and lack the potential to simulate the function of microorganisms [16].

In adjusting N-dynamics, the organic component of soil and its labile portions play a significant role in determining available N for plants and N mineralization [14,17]. Mineralization of the organic component of soil mainly increases N availability of soil and meets between 50 and 80% of plants’ N requirements [12,18]. The upper layer of soil is considered to have the highest soil organic N, i.e., about 95% of the overall N [19]. The soil organic N moves in the soil over ground crop litter and root litter as particulate organic matter [14]. The particulate organic matter may offer further precise details on N mineralization as it includes simply mineralizable N portions. This portion characterizes the mineralizable pool and it offers a simple and feasible way to estimate the N mineralization capacity [14]. Haynes [20] stated that particulate organic matter is a significant labile N pool in numerous soils. Nevertheless, residue input and climate situations have a strong impact on the decomposition of this labile portion [14], which changes over years in semi-arid and semi-humid regions.

There is significant uncertainty regarding the function of mineralization indices or SOM portions in the N-cycle, and they have not been confirmed as a soil measurement technique to identify N [11]. In contrast, the N mineralization could be influenced by the chemical and physical characteristics of the soil [12]. Nevertheless, our skill to estimate N mineralization may be enhanced by recognizing their role, as well as by studying their interactions with other soil characteristics in the N-cycle. Therefore, the estimation of N mineralization in various soils could be enhanced by incorporating these characteristics in multiple regression (MR) models [11,21]. Several investigations have linked soil characteristics and climate situations to N mineralization [21]. The precision of estimation of potential N mineralization could be enhanced by merging SOM portion and soil characteristics in MR models [21,22,23]. Nevertheless, several researchers [11,24] have stated that there is a potential cause of multicollinearity between the model’s factors, and that the biological logic of variables in their mineralization procedure may be complicated by applying MR. One technique to address the multicollinearity and specify the interaction of all variables in various dimensions is to apply a statistical method that can categorize very correlated parameters into meaningful data and can be applied as a new category of independent variables [24]. Currently, there are some related investigations via associating of N mineralization indices, labile organic portions, and soil characteristics by involving two or more variables in analyses. Moreover, to estimate the potential N mineralization, this statistical analysis incorporates multivariate methods such that the major soil parameters contributing to model fit can be specified [11,25,26]. Furthermore, Martínez-Lagos et al. [18] stated that enhancing suitable fertilization approaches requires having data on the cycling and the size of the inorganic-N pool. 

Several studies have been conducted on soil sensors development and enhancement methods in order to measure soil properties [27,28,29,30,31,32,33,34,35]. The mechanism of these approaches was built on electronics, mechanics, and chemistry aspects of the soil [36,37,38]. Chighladze et al. [39] studied two sensors (ECH2O EC-5 and EC-10) to evaluate their response on availability of different concentrations in soil (e.g., NO_3_, Cl). Their results revealed that the sensor’s response to difference rates of NO_3_ were more sensitive than variations in Cl. Furthermore, they stated that high-frequency and low-frequency assessments can be applied to evaluate soil moisture content and some concentrations in soil (e.g., salinity and different ions), respectively. 

To monitor changes in soil physical and chemical properties, soil EC maps can be utilized [40]. Many studies have highlighted the inevitable relationship between soil EC with changes in soil physical and chemical properties, and crop productivity [41,42,43,44,45,46,47,48,49,50,51,52,53]. Grisso et al. [54] stated that soil characteristics are correlated with soil EC. They specified that soil cation exchange capacity (CEC), topsoil depth, salinity, texture, organic matter, pH, and moisture content have an influence on soil EC. Furthermore, soil EC maps can be applied for precision agriculture decisions and site-specific crop management. Zhang and Wienhold [55] studied the impact of changes in soil moisture content on EC, soil mineral N, and pH in a research laboratory condition. The study reported that an increase in soil moisture level in the range of 0–0.008 m^3^ resulted in an increase in soil NO_3_^−^ N, however, increasing soil moisture above this level did not result in variation in NO_3_^−^ N but raised NH_4_^+^ N. There was no variation in soil pH with respect to increased soil moisture. The study found a positive correlation (with a portable EC sensor, R^2^ = 0.85; in the laboratory as 1:1 soil water slurries R^2^ = 0.92) between soil EC and soil mineral N. Authors recommend using soil EC for quick identification of variations in soil inorganic N rate in soils with lower salt and carbonate-free conditions.

Recently many investigators have attempted to develop EC sensors and to enhance techniques for soil EC measurement [56,57,58,59]. They have used EC sensors to monitor soil properties due to their easy and fast soil measurement. For instance, EC sensors have been used to monitor soil salinity, estimate plants’ water requirements, and improve irrigation systems [60,61]. An EC sensor was used to evaluate the impacts of existing waste on soil chemical properties and crop development [62,63]. It is also feasible to monitor some soil characteristics and the spatial distribution of clay using soil EC [64]. Therefore, soil electrical conductivity can be used for site-specific management by categorizing areas with close soil properties faster and more inexpensively. Nevertheless, several soil properties such as moisture content, organic matter, salinity, texture, soil mineralization, density, and temperature, affect soil electrical conductivity [65,66,67,68]. Moreover, studies have demonstrated that soil characteristics with a larger impact on soil electrical conductivity should be taken into account for investigated fields [69,70]. Small and marginal farmers often face problems with variability in their fields and cannot afford costly and sophisticated methods for getting information about variability and applying input accordingly. There is a need for an innovative, smart, and cost-effective sensing system. Electrical conductivity-based soil moisture and available N sensors could potentially bridge this gap. However, a key prerequisite for this advance is a clear understanding of the interactions between EC and soil moisture, as well as between EC and available N for different soils with varying moisture levels. Thus, in view of the above, the present study seeks to address this gap in order to develop a sensor that can form the base for any type of variable rate detection using electrical conductivity for site-specific N application by exploring the relationships between electrical conductivity, moisture content, and N level for different selected soils in the field. Based on the relationships observed, an algorithm was developed for electrical conductivity and moisture-content-based soil N management.

## 2. Materials and Methods

The experiments in this study were conducted in two phases at the division of Agricultural Engineering, Centre for Protected Cultivation Technology, the division of Agronomy, the division of Soil Science and Agricultural Chemistry, and the division of Agricultural Physics, IARI, New Delhi. In the first phase, an experiment was carried out to characterize the soil samples in terms of soil texture. Samples from five types of soils were selected from IARI fields and Yamuna river banks to form a representative sample of all major soil types in India. These five different soil types were analyzed for their textural characteristics and, accordingly, three soil types representing a soil with maximum clay, sand, and silt content, respectively, were selected for further experimentation. Samples from these three soil types were then analyzed for their physical and chemical properties using standard methods. 

### 2.1. Measurement of Soil Physical Properties

#### 2.1.1. Soil Texture

Soil texture was determined using the Bouyoucos hydrometer method [71]. Two beakers were taken and one was filled with 0.050 kg of the dry soil sample. Sodium hexametaphosphate (NaPO_3_)_6_ weighing 0.100 kg was mixed with 1000 mL distilled water and 50 mL of sodium hexa meta phosphorus solution was added to the soil sample and shaken well. An amount of 100 mL of distilled water was then added and stirred and the solution was kept at room temperature for 4–5 h. The samples were then transferred to a larger cylinder and distilled water was added to each soil sample until the total volume of the solution became 1000 mL. The cylinder was shaken vigorously several times so that soil particles dispersed completely. Then, the hydrometer was immediately placed in the suspension and data was collected after 40 s. After 2 h, an additional hydrometer reading was taken. All the experiments were repeated without the soil sample, i.e., repeated with water and were denoted as blank.

The percentage of silt and clay was calculated using the following formula [72], initial reading.

$$\mathrm{percentage}\;\mathrm{of}\;\mathrm{silt}\;\mathrm{and}\;\mathrm{clay}=\frac{\left(\mathrm{Soil}\;\mathrm{sample}\;\mathrm{reading}-\mathrm{Blank}\;\mathrm{reading}\right)+\mathrm{CF}}{\mathrm{weight}\;\mathrm{of}\;\mathrm{Soil}\;\left(\mathrm{kg}\right)}\times 100$$

CF = Correction Factor = (T°F − 68) × 0.2

Second reading after 2 h

$$\mathrm{percentage}\;\mathrm{of}\;\mathrm{clay}=\frac{\left(\mathrm{Soil}\;\mathrm{sample}\;\mathrm{reading}-\mathrm{Blank}\;\mathrm{reading}\right)+\mathrm{CF}}{\mathrm{weight}\;\mathrm{of}\;\mathrm{Soil}\;\left(\mathrm{kg}\right)}\times 10$$

#### 2.1.2. Field Capacity

A few days after wetting of the soil, when the water has drained, the volume of water maintained in the soil is the field capacity [73]. The field capacity was computed using a dash pressure extractor. Soil samples were collected in volumetric rings, which were saturated on a ceramic plate for one night. The saturated soil-filled rings were then put inside the dash pressure plate apparatus. Water was drained out of the soil by maintaining a pressure of 22 kPa in the apparatus for one week. After one week, the moisture content of the soil sample was measured using the oven drying method [74]. The resulting moisture content was the field capacity (FC).

#### 2.1.3. Permanent Wilting Point 

Permanent wilting point was evaluated by using a dash pressure extractor as was done for field capacity. Soil samples were collected in volumetric rings and were saturated on a ceramic plate for one night. The saturated soil-filled rings were then put inside the dash pressure plate apparatus. Water was drained from the soil by maintaining a pressure of 100 kPa in the apparatus for one week. After one week, the moisture content of the soil sample was measured using the oven method. This moisture content was the permanent wilting point (PWP) [73].

#### 2.1.4. Permanent Available Water Capacity

The volume of water that can be used by crop roots is the total available water capacity [73]. Available water capacity (AWC) was calculated by subtracting the moisture content at permanent wilting point from that at field capacity.

### 2.2. Determination of Chemical Properties of Selected Soil Types

The chemical properties of soil, such as pH, available N and total N, organic carbon (OC), phosphorus, and potassium, were determined.

#### 2.2.1. Soil pH

Soil pH was determined using the KCL method [75]. Fifty ml of 1 molar (M) KCL solution was poured into a beaker (100 mL capacity) filled with 0.020 kg of soil sample. Suspension was stirred for 15 min and pH was determined on a pH meter using a glass electrode.

#### 2.2.2. Total N of soil

For determination of total N in soil, the Kjeldahl digestion method was used [76]. Organic N in the soil sample was converted to NH_4_-N by digestion with concentrated H_2_SO_4_ containing substances (K_2_SO_4_ or Na_2_SO_4_) that promote this conversion. The NH_4_^+^ N in the digestion was determined from the amount of NH_3_ liberated by distillation of the digest with an alkali (sodium hydroxide) (NaOH). Soil sample, weighing 0.005 kg which was passed through a 100-mesh sieve, was transferred to a 500 mL Kjeldahl flask. An amount of 25 mL of concentrated H_2_SO_4_ was added and allowed to stand for 30 min. Then, 0.005 kg of sodium thiosulphate was added, and it was again allowed to stand for 30 min. A catalyst mixture was added and heated in a Kjeldahl flask; at first, it was heated slowly until frothing began, and then was heated rapidly. Digestion continued for half an hour. It was then cooled and combined with 150 mL of water. One hundred twenty ml of 40% NaOH solution was applied along the sides of the Kjeldahl flask. Distilled ammonia evolved in 25 mL of boric acid solution was collected continuously until 150 mL of distillate was obtained. Titration of ammonia was done against standard sulfuric acid. A blank sample test was run simultaneously with a piece of paper (of the same size as those used for wrapping the samples) and other reagents.

$$\mathrm{Total}\;N\;(\%)=\frac{\left(\mathrm{mL}\;\mathrm{of}\;\mathrm{acid}\;\mathrm{used}\;\mathrm{for}\;\mathrm{sample}\;-\;\mathrm{mL}\;\mathrm{of}\;\mathrm{acid}\;\mathrm{used}\;\mathrm{for}\;\mathrm{blank}\right)\;\times \;0.02\;\times \;14\;\times \;10^{-3}}{\mathrm{Sample}\;\mathrm{weight}\;\mathrm{in}\;\mathrm{kg}}\;\times \;100$$

#### 2.2.3. Available N in Soil

Potentially available N in soil was estimated using calcium hydroxide hydrolysis, and the NH_3_ liberated was absorbed in boric acid containing a mixed indicator. The amount of NH_3_ was calculated by titration of distillate against standard acid (HCl or H_2_SO_4_). A 0.020 kg of soil sample was transferred into a 500 mL Kjeldahl flask and combined with a quarter tea-spoon of calcium hydroxide dissolved in 200 mL water plus a few glass beads, and a drop of a heavy mineral oil. Ammonia was collected by connecting the flask to the Kjeldahl distillation assembly in a 250 mL Erlenmeyer flask, which contained 25 mL of 4% boric acid mixed indicator solution placed below the receiver. Distillation continued for 30 min. Finally, ammonia was titrated and collected against a standard sulfuric acid solution and a blank sample was run simultaneously.

Available N was calculated using the formula blow:

Available N (kg ha^−1^) = (mL H_2_SO_4_ used for sample − mL H_2_SO_4_ used for blank) × Normality of H_2_SO_4_ × 1568 *

$$*\;(\frac{\mathrm{Weight}\;\mathrm{of}\;0–0.15\;m\;\mathrm{layer}\;\mathrm{of}\;\mathrm{soil}\;\mathrm{in}\;\mathrm{kg}}{\mathrm{Sample}\;\mathrm{weight}\;\mathrm{in}\;\mathrm{kg}\;}\;\times \;\mathrm{Equivalent}\;\mathrm{weight}\;\mathrm{of}\;N=\frac{2.24}{0.02}\times 14=1568)$$

Experimental procedure for study of effect of soil moisture and N level on electrical conductivity of selected soil types.

Experiments were conducted to measure electrical conductivity of different soil types at varying levels of soil moisture and N as per the experimental layout, Table 1.

### 2.3. Measurement of Soil Electrical Conductivity

The capacity of an element to transfer a current is electrical conductivity (EC). It is generally known in milliSiemens per meter (mS m^−1^) units. It is sometimes expressed in deciSiemens per meter (dS m^−1^) units as well, and equals to 0.01 mS m^−1^ [77]. An EC sensor (HI98331 Soil Test™ direct soil EC Tester, Hanna Instruments, Woonsocket, RI, USA) was used to measure soil electrical conductivity. The sensor consisted of two metal electrodes and a constant voltage was applied across the electrodes resulting in an electrical current flowing through the sample.

#### 2.3.1. Measurement of EC at Different Levels of Soil Moisture

Five levels of moisture content of different soils were included in the study. The five levels of moisture content were 0% (dry soil), 13%, 15%, 17%, and 22% (dry basis). A moisture content of 22% was considered as field capacity [78] and 13% represented ploughing moisture [79]. Therefore, one level was initial soil moisture, which was zero moisture or dry soil, and for others, the moisture levels constituted the base for choosing various levels. To achieve the required moisture content in the soil, a calculated amount of water was added to dry soil. The selected levels of moisture content were obtained with the help of a moisture sensor (HI98331 Soil Test™ direct soil EC Tester, Hanna Instruments, Woonsocket, RI, USA) as well as by dry oven method testing. Electrical conductivity of different soils with different levels of moisture content was directly measured in miliSimon per meter using a soil EC sensor.

#### 2.3.2. Measurement of Soil EC at Different Levels of N

Four levels of N 50 kg, 100 kg, 150 kg, and 200 kg ha^−1^ were added to the soil by adding a calculated amount of urea and then the electrical conductivity at each level was measured with an EC meter. A 0.100 kg soil sample of each soil type was used in this experiment. Soil weight from 0 to 0.15 m of depth was considered as 2.2 × 10^6^ kg ha^−1^ [80] and, accordingly, the level of N in the soil was adjusted by adding urea (46% N). A sample of 22 mL of water was mixed with different amounts of urea and added to 0.100 kg of soil sample so as to achieve a constant moisture content of the soil, i.e., 22%. Using an EC meter, the electrical conductivity of N-enriched soil was measured at a constant moisture level of 22%. The same procedure was followed to measure soil EC for other levels of soil moisture and N levels.

#### 2.3.3. Statistical Analysis

To evaluate the effect of individual and interactive effects of independent variables on the response variable, an analysis of variance was carried out for a randomized block design. The analysis of variance (ANOVA) was carried out individually for the significance of moisture and N on electrical conductivity.

## 3. Results

### 3.1. Physical Properties of Selected Soil Types

The results for various physical properties of selected soil types are presented below.

#### 3.1.1. Soil Texture of Selected Soil Samples

In all the studied representative soil samples, sand was predominantly present and there was also a distinct variation in clay percentage. The clay, silt, and sand percentage ranged between 16–45.84%, 18–41.32%, and 36.16–58.52%, respectively (Table 2). Out of five samples, three soil samples were selected for further study based on the maximum percentages of sand, clay, and silt.

#### 3.1.2. Field Capacity, Permanent Wilting Point, and Available Water Capacity 

A maximum field capacity of 23.63% was observed for the clay soil sample followed by 18.21%, which was observed for a soil sample higher in sand (61% sand, Table 3). This aligns with the findings of a previous study conducted by Saxton et al. [81], which also showed that the maximum field capacity was observed for soil with 50% clay and 25% sand content. The soil sample with the highest silt percentage exhibited the minimum permanent wilting point due to having less clay content which is similar to the conclusions drawn by Saxton et al. [81]. The minimum and maximum permanent wilting points were 5.66% and 12.23%, respectively. The available water, computed as the difference of the above two variables, showed the expected pattern, i.e., the amount of available water was highest for the soil with the highest clay percentage. In general, it was observed that clay loam soil exhibited the highest field capacity, permanent wilting point, and available water capacity due to having a higher percentage of clay and less porous space. The role of clay percentage in determining these characteristics was important. A summary of soil characteristics and composition is included below (Table 3).

### 3.2. Chemical Properties of Selected Soil Types

#### Nitrogen in Soil

The available N in kg ha^−1^ was highest (112.1 kg ha^−1^) in sandy loam soil with a higher sand proportion followed by clay loam (94.1 kg ha^−1^) and sandy loam (37.1 kg ha^−1^), which had a higher silt percentage. The total N percentage revealed a similar pattern for the three different soils. The total N percentage ranged from 0.01 to 0.07%, and was the highest for sandy loam soil with a higher sand percentage (Table 4).

### 3.3. Variation in Electrical Conductivity with Moisture Levels

**Figure 1:**
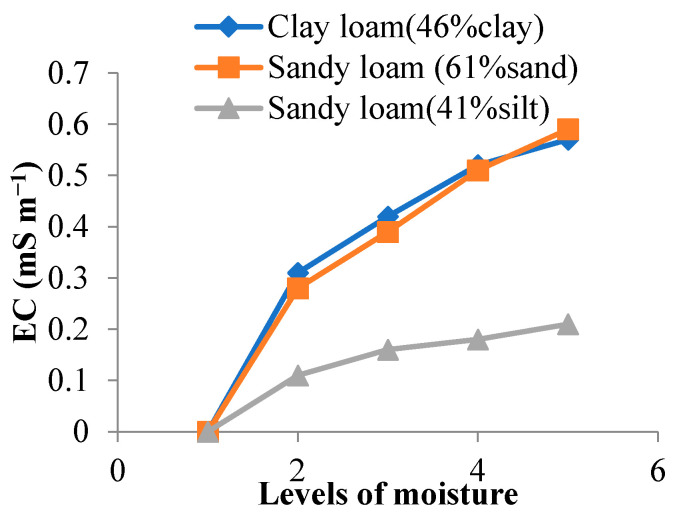
Relation between moisture and EC for different soil samples.

The EC of all three soils was found to increase as moisture levels increased. The highest EC of 0.59 mS m^−1^ was observed for sandy loam soil (61% sand) followed by 0.57 mS m^−1^ for clay loam soil (46% clay). The lowest EC value of 0.21 mS m^−1^ was observed for sandy loam soil (41% silt) at all the studied soil moisture levels ranging from 13–22%. The result showed 0.00 mS m^−1^ for dry soil, which increased with increases in moisture content. The EC of sandy loam, with 61% sand, was the highest as this soil sample had the maximum available N and total N (Figure 1).

For clay loam, the increase in EC was 83.87% when moisture was increased from 13 to 22%. Likewise, the same difference in EC was observed as moisture level was increased for both sandy loam with higher silt and sandy loam with higher sand; their corresponding EC increases were 91% and 111%, respectively. Therefore, moisture level significantly influenced the EC of all three soils. 

### 3.4. Variation in Soil Electrical Conductivity by N Level

**Figure 2:**
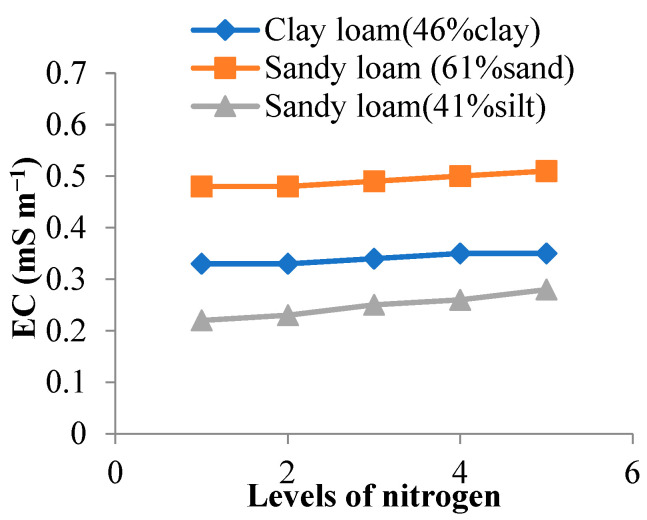
Relation between N and EC for different soil samples.

The EC of three different types of soils ranging from sandy loam to clay loam revealed an increasing trend with the level of N gradually increasing from 50–200 kg ha^−1^ (Figure 2). EC was highest (0.51 mS m^−1^) for sandy loam (61% sand) and lowest (0.22 mS m^−1^) for sandy loam (41% silt) soil, as demonstrated in Figure 2. For clay loam, the increase in EC was 6.1% when the N level was increased from 50 kg ha^−1^ to 200 kg ha^−1^. For sandy loam (61% sand), the EC increased by 6.2% with an increase in N level from 50 kg ha^−1^ to 200 kg ha^−1^, while for sandy loam (41% silt), the change in EC was the highest (21.7%) for the same change in N level. Thus, the influence of N on EC was found to be the highest in soil with the maximum silt percentage.

### 3.5. Algorithm for Real Time N Measurement

An algorithm was developed using a polynomial relationship between the EC of the selected soil samples and their moisture levels. A polynomial relationship was also developed between the EC of each of the selected soil samples and their N levels. A significant correlation was observed between EC and different levels of N as well as different levels of moisture. The equations of this algorithm are as follows:

(A) For different levels of moisture,
  (i). For clay loam (46% clay)
    y = −0.037x^2^ + 0.362x − 0.306 (R^2^ = 0.985)
  (ii). For sandy loam (61% sand)
    y = −0.027x^2^ + 0.308x − 0.264 (R^2^ = 0.988)
  (iii). For sandy loam (41% silt)
    y = −0.013x ^2^ + 0.130x − 0.11 (R^2^ = 0.981)
  where (y) is EC of soil and (x) is the moisture level (%) and R is regression coefficient
(B) For different levels of N,
  (i). For clay loam (46% clay)
    y = 0.0014x^2^ − 0.0006x − 0.478 (R^2^ = 0.983)
  (ii). For sandy loam (61% sand)
    y = 0.006x − 0.322 (R^2^ = 0.900)
  (iii). For sandy loam (41% silt)
    y = 0.0007x^2^ + 0.0107x + 0.208 (R^2^ = 0.99)
  where (y) is EC of soil and (x) is level of N and R is the regression coefficient.

The coefficients of determination for N levels were 0.98, 0.90, and 0.99 for clay loam (46% clay), sandy loam (61% sand), and sandy loam (41% silt), respectively.

The data showed that when input into the equation that resulted from the correlation between EC and nitrate-N soil concentration, EC values provided reliable information about N availability. However, there is need to further investigate the interference of factors such as soil texture, CEC, salinity, pH, and organic matter.

## 4. Discussion

Nitrogen is the main element used in plant fertilization and measurement of its concentration in soil yields reliable information on N availability. Electrical conductivity indicates the presence of the major inorganic solutes dissolved in the aqueous phase consisting of soluble and readily dissolvable salts in soil solution, which include cations (e.g., Na^+^, K^+^, Mg^2+^, and Ca^2+^), anions (Cl^−^, HCO_3_^−^, NO_3_^−^, and SO_4_^2−^), and nonionic solutes [69]. Thus, soil EC has become one of the most frequently used measurement methods for characterizing nutrient variability in the field for application in precision agriculture [82]. The data revealed that the values of EC provided useful information on soil N availability using the equation that resulted from the correlation between EC and nitrate-N soil concentration. In this study, the relationship between soil EC and N availability for all the soil types was best described using a second degree. Similar studies conducted in controlled conditions [83,84] as well as in field experiments [85,86,87] have found a direct relationship between EC and nitrate concentration in soil solutions. However, there is need to further study if and how additional soil characteristics may interact with this relationship.

We observed that the soil EC changed across soil textures in accordance with previous studies in which a correlation between EC and soil texture was found [88,89]. The EC of different soils ranged between 0.00–0.59 mS m^−1^ for different soil moisture content of between 0–22% and EC increased with an increasing moisture content of soil. This correlation between EC and soil moisture content was observed in previous studies. However, in those studies, Faulin and Molin [90] concluded that there was a significant correlation between soil EC and moisture content, while Valente et al. [41] stated that there was no significant correlation between soil moisture content and EC. 

In this study, results showed a significant correlation between EC with sandy loam soil (61% sand) and clay loam soil (46% clay) which is similar to previous studies’ results. Specifically, [70,89,91,92] reported a significant relationship between EC and soil with clay content. However, other studies [40,93,94,95,96,97] reported this correlation was not significant, underscoring the need for further research on this topic. This dissimilarity across studies could be due to differences in the combined percentage of clay, sand, and silt of the soils being tested, or may stem from variations in soil inorganic N rate in those soils [59].

The results of changes in EC with change in N levels revealed a correlation between soil EC and soil N, and soil EC was increased in different soil samples by increasing soil N; this correlation has been reported in previous studies as well [59,98].

The prediction models presented in this study concern three soil types: clay loam (higher clay), sandy loam (higher silt), and sandy loam (higher sand), which paved the way to explore similar trends in other soil types which have intermediate soil texture. However, there should be future studies on many other different soil types. Moreover, in the field, reality might be complex (due to dynamic factors such as climate or the impact of other physical and chemical properties of soil that are not considered in this study). Therefore, further research should be conducted on field conditions considering other physical and chemical properties of soil as well.

## 5. Conclusions

Samples of three soil types were selected based on maximum percentages of sand, clay, and silt present in each with the goal of developing an understanding of the relationships between soil EC, moisture content, and N level. The nitrogen content and moisture content in all selected soil types was found to significantly affect the EC of the soil. Therefore, accurate information regarding the EC and texture class of soil can be used to inform how its nitrogen content can be optimally managed. The relationships established can be used to develop a sensor for real-time measurement of available soil N, which, in turn, will enable farmers to precisely apply nitrogenous fertilizer.

## Figures and Tables

**Table 1:** Plan of experiment for determining relationships between EC, soil moisture, and N level of soil.

Variables	Levels	Parameter Measured
Soil texture	Clay loam (maximum clay percentage)	Electrical conductivity
	Sandy loam (maximum silt percentage)	
	Sandy loam (maximum sand percentage)	
Soil moisture, percent (dry basis)	0 (dry soil)	Electrical conductivity
	13	
	15	
	17	
	22	
N Level, kg ha^−1^	0 (no addition of N in soil)	Electrical conductivity
	50	
	100	
	150	
	200	

**Table 2:** Percentage distribution of sand, silt, and clay in selected soil types.

Percentage of Sand Silt and Clay (%)				
Soil Type	Clay	Silt	Sand	Selected Based on
1	27.12	20.0	52.88	-
2	21.12	21.12	57.76	-
3	9.12	30.0	60.88	Sand content
4	45.84	18.0	36.16	Clay content
5	0.16	41.32	58.52	Silt content

**Table 3:** Physical properties of selected soil types.

Soil Properties	Clay Loam (46% Clay)	Sandy Loam (41% Silt)	Sandy Loam (61% Sand)
Field capacity (%)	23.63	13.96	18.21
Permanent wilting point (%)	12.23	5.66	7.28
Available water capacity (%)	11.4	8.3	10.93

**Table 4:** Total and available N in different soil.

Type of Soil	Clay Loam (46% Clay)	Sandy Loam (41% Silt)	Sandy Loam (61% Sand)
Total N (%)	0.01	0.04	0.07
Available N (kg ha^−1^)	94.1	37.1	112.1

## Data Availability

Not applicable.
